# Self-reported psychological disorders among the mothers of children with autism spectrum disorder, type 1 diabetes mellitus, and typically developed children

**DOI:** 10.1186/s11689-021-09369-y

**Published:** 2021-05-22

**Authors:** Ahmed Malalla Al Ansari, Mohamed Ismael Janahi, Abdulrahman J. AlTourah, Haitham Ali Jahrami, Mansour Bin Rajab

**Affiliations:** 1 College of Medicine and Medical Sciences, Arabian Gulf University, Manama, Kingdom of Bahrain; 2 Governmental Hospitals, Ministry of Health, Manama, Bahrain; 3 Department of Pediatrics–Salmaniya Medical Complex, Ministry of Health, Manama, Bahrain

**Keywords:** ASD, Diabetes, DASS-21, PSS-14, Bahrain

## Abstract

**Background:**

To assess the prevalence of symptoms of depression, anxiety, and stress among mothers of children with autism spectrum disorders (ASD), type 1 diabetes (DM), and typical development (TD), in a geographical area where such data are lacking

**Method:**

A descriptive study with the three groups of parents of children with and without a condition was conducted (ASD n=126, group 1; DM n=43, group 2; and TD n= 116, comparative group). Measures of depression, anxiety, and stress were collected to examine the prevalence of factors, difference between groups, and their association with demographic characteristics.

**Results:**

On the DASS-21, both groups 1 and 2 had higher mean scores for depression (37.86), anxiety (4.58), and stress (29.81) than the control group (P=0.015). On the PSS-14, the mean score was higher in group 2 (28.63) than in group 1 (27.61) and the comparison group (25.87) (P=0.004). On the DASS 21, group 1 scored higher in the depression domain (P=0.046), whereas group 2 scored higher in the anxiety domain (P=0.034) and stress domain (P=0.009) than the TD group.

**Conclusion:**

Mothers of children with ASD should be assessed for the presence of depression following diagnosis. Mothers of children with type 1 diabetes require careful monitoring for the effects of anxiety and stress on their mental health and therefore their ability to cope with diabetes management plans.

**Trial registration:**

Not applicable.

## Background

Several studies have reported that families of children with autism spectrum disorder (ASD) experience more parenting stress than families of children with typical development (TD) or those diagnosed with other disabilities [1–4]. From these studies, an emerging trend suggests that mothers of children with ASD exhibit higher levels of depression, anxiety, and stress than the general population.

Several international studies support what has been reported previously. A study in Italy assessed the parental burden in families of children with ASD compared to a control group of parents with children who were diagnosed with type 1 diabetes mellitus and parents with children diagnosed with Down syndrome. The parents of children with ASD reported higher objective and subjective burdens, more frequent psychological distress, and lower social support. Moreover, mothers reported a greater subjective burden than fathers [5]. A study in the Netherlands found that both parents reported more stress when parenting their child with ASD and/or ADHD than when parenting the unaffected sibling; they also experienced more stress than the general population [6]. In the USA, a multistate-wide study reported that parents of toddlers with ASD demonstrated increased parenting-related stress compared with parents of toddlers with developmental delay and typically developed children. However, psychological distress did not differ significantly between the groups [7]. Although parental stress, anxiety, and depression were reported to be significantly higher in parents of children with ASD than in parents of children with TD, a study in the UK found that parent-reported distress, anxiety, depression, and health were not correlated with physiological measures, with the exception that parents of children with ASD had significantly lower cortisol levels 30 min after waking [8].

Studies that addressed the prevalence of depression, anxiety, and stress among mothers of children with diabetes have not been conclusive and reported mixed results. Parents of children with type-1 diabetes have a higher expression of anxiety and depressive feelings than parents of children without diabetes [9–12]. All subjects in the above studies included both parents. A study by Hauenstein et al. several years ago [13] did not find a difference in a group of mothers regarding their overall stress. Another study reported considerable anxiety among mothers of children with type 1 diabetes, but anxiety was not associated with child’s metabolic control [14]. The degree of anxiety and stress was not only high in the period following diagnosis but worries continued for a longer period due to the chronic nature of the illness [15].

Similar findings have been revealed in the EMRO region. One study in Oman revealed that caregivers of children with ASD carry a greater risk of depression and stress than caregivers of children with intellectual disabilities, along with an even greater risk when compared to the general population [3]. A study in Saudi Arabia found mean scores of depression and anxiety that were significantly higher in a sample of parents of children with ASD than in a control sample consisting of parents of children with TD [4]. A study from Iran noted that the most common psychiatric conditions among parents of children with ASD were poor mental health including “melancholic personality, persistent depression, and negativism” [16]. A cross-sectional study in Kuwait found that mothers of children with ASD had higher levels of parenting-related stress and psychological distress than controls. The study also reported no significant difference between fathers of children with ASD and fathers from the general population [17]. In Bahrain, mothers were found to be negatively impacted by having children with ASD. The sample studied was found to have more physical and mental health issues, along with a decreased quality of life, than the general population. Mothers also reported more physiological and environmental problems than mothers of children with intellectual disability and typically developed children [2, 18]. We could not identify any local study addressing the mental health of mothers of diabetic children.

The goal of this study is to identify any significant differences in depression, anxiety, and stress among mothers of children with ASD when compared to mothers of children with another chronic illness, diabetes, and mothers of children with typical development (TD). The study will provide information on the mothers’ health with respect to both ASD and diabetes in a geographical area where such information is lacking.

Type 1 diabetes was chosen as the chronic illness under investigation, due to its presumed high impact on mother’s mental health. The study hypothesis was that the psychological distress among mothers of children with ASD would be higher than mothers of children with type 1 diabetes or TD children.

## Method

### Design

This was a case-control study conducted in Bahrain; participants were recruited from autism centers and diabetic clinics.

### Sample

The cases consisted of 126 mothers of children aged between 3 and 18 years with a diagnosis of ASD (group 1) or type 1 diabetes (group 2). The diagnosis of ASD was clinically reached using the Modified-Checklist for Autism in Toddlers (M-CHAT), Diagnostic and Statistical Manual for Mental Disorders 5 (DSM-5) criteria, Childhood Autism Rating Scale (CARS), and Autism Diagnostic Observation Schedule (ADOS) with certain individuals at the Child Psychiatric Unit, Psychiatric Hospital, Kingdom of Bahrain [19]. The number of potential participants was initially identified as 172, which was later reduced to 126, as 46 individuals were excluded due to non-eligibility. The inclusion criteria for participation were mothers who had one child aged 3–18 years with either ASD or type 1 diabetes. The exclusion criteria were mothers who had other children with a disability or chronic illness defined as continuous symptoms for more than 6 months and mothers who had legal, marital, or financial difficulties; mental health diagnoses; or chronic physical problems in the month before the initial interview. Seventy-seven mothers of children diagnosed with type 1 diabetes were recruited from the Pediatric Department, Outpatient Clinic, Salmaniya Medical Complex, which was later reduced to 43 based on the same exclusion criteria. These exclusion criteria were selected to reduce the effects of confounding stresses and to obtain a more pure look at the distress. All individuals attending the autism centers and the diabetes clinic between June 15 and October 15, 2019, were recruited using convenience sampling.

### General population control

Mothers from the general population that matched groups 1 and 2 both in the mean age of the child and family size were randomly recruited. The control group included mothers who had a child without any neurodevelopmental disorder or chronic illness. A chronic illness was defined as an illness that lasted continuously over 6 months and is known to have an impact on the mother’s mental health. The same exclusion criteria used for groups 1 and 2 were applied to the control group (*n*=116).

### Procedures

Members of the research team (A.T. and M.J.) contacted all eligible individuals by electronic means. Sixty mothers from group 1 were interviewed face-to-face due to a lack of computer literacy. All mothers in groups 1 and 2 and the control group completed both the DASS-21 and PSS-14 (Arabic versions) questionnaires. The mothers were informed verbally of the nature and the objectives of the research, and their approval to participate in the study was obtained. It was explained to all participants that enrollment in the study was voluntary and would not affect the services provided to them and their children.

### Instruments

A datasheet to record the child’s and mother’s age, employment, and education was used along with a validated Arabic version of the DASS-21 which is a standard assessment tool for depression, anxiety, and stress symptoms, with several items focused on each of the three dimensions. These items directed the recipient to describe only their experiences during the previous week. This tool has been reported to have acceptable validity for stress (r=0.74) and anxiety (r=0.81) when compared to the Beck Depression Inventory [20]. The Arabic version of the DASS-21 has a Cronbach alpha of 0.88 [21]. The validated Arabic version of the Perceived Stress Scale (PSS) is the most widely used psychological instrument for the measurement of one’s perception of their stress. For the PSS, the recipients were asked to describe their thoughts and feelings during the last month [22]. The Arabic version of the DASS-21 has a Cronbach alpha of 0.75 [23].

### Data analysis

Data were entered into SPSS version 26. Descriptive statistics were summarized for the demographic characteristics and outcome measures. The means and standard deviations (SD) are reported for continuous variables, and applicable counts and percentages are reported for categorical variables. Pearson’s chi-square or Fisher’s exact tests and independent-sample t-tests were used to investigate the differences among the groups. Odds ratios and their respective confidence intervals were also determined for the DASS-21 measures of the severity of stress, anxiety, and depression between groups 1 and 2 and the TD-control group. Multiple logistic regression analysis was performed to examine the association between self-reported depressive symptoms, anxiety, and stress and predictive variables such as maternal education and employment.

## Results

Table 1 shows the demography of individuals in groups 1 and 2 and the control group. There were no differences in mean child age, the mean number of children, or the mother’s age group (31–60 years) between the groups. There was no difference between the diabetes group and the control group in terms of education, but mothers in the control group were employed significantly more often than those in the diabetes group. On the other hand, mothers in the ASD group were significantly less educated and less employed in comparison to the TD-control group. Results from multiple logistic regression analysis showed that maternal education and employment are significant difference between groups.

**Table 1:** Mother’s age, mother’s education, and mother’s employment in the ASD, diabetes, and control groups

Variable	ASD group n	%	Diabetes group n	%	Control group n	%	*P* value
**Child age (mean)**	8.26		9.8309		8.6474		0.571
**Number of children (mean)**	3.1		3.09		2.77		0.071
**Mother’s age (y)**							.382
20–30	34	27	8	18.6	34	29.3	
31–60	92	73	35	81.4	82	70.7	
**Mother’s education**							.007
Reading and writing	6	4.8	1	2.3	0	.00	
Primary/middle school	16	12.7	1	2.3	3	2.6	
High school	42	33.3	19	44.2	42	36.2	
University	62	49.2	22	51.2	71	61.2	
**Mother’s employment**							.000
Working	32	25.4	15	34.9	68	58.6	
Homemaker	74	58.8	23	53.5	44	38.0	
Retired	19	15.1	5	11.6	4	3.4	
Student	1	0.9	0	.00	0	.00	

Table 2 shows the mean DASS-21 and PSS-14 scores among the diabetes group, group 2, and the control group. Total DASS-21 scores were higher in group 2 than in the community controls (41.58 vs 29.81; P=0.019). The DASS-21 subscale mean scores for depression and stress were higher in group 2 than in the control group, but the difference did not reach significance. The PSS-14 mean scores were significantly higher in group 2 than in the control group (28.63 vs 25.87; P=0.003).

**Table 2:** DASS-21 and PSS-14 mean scores between the diabetes group and control group

Instrument	Diabetes group mean score *N*=43	Control group mean score *N*=116	*P* value	Odds ratio	95% confidence interval	
**DASS-21 total**	41.58	29.81	0.019			
**Depression**	10.81	9.34	0.395	1.148	0.568	2.318
**Anxiety**	10.91	7.81	0.053	2.228	1.085	4.573
**Stress**	14.05	12.66	0.404	1.324	0.645	2.717
**PSS-14**	28.63	25.87	0.003			

Table 3 shows the mean DASS-21 and PSS-14 scores for the mothers of children with ASD (group 1) and diabetes (group 2). On the DASS-21, ASD group scores were higher for depression and stress than those for the diabetes group, while the diabetes group scored higher for anxiety symptoms (OR=1.742, 95% CI=0.856–3.444, P=0.642), although this difference did not reach a significant level. On the PSS-14, the diabetes group scored higher on the stress measure than the ASD group, but again, the difference was not significant (28.63 vs 27.61; P=0.245). The DASS-21 total scores of all measures were higher for group 2 (41.58) than for group 1 (37.86), but the difference did not reach significance.

**Table 3:** DASS-21 and PSS-14 mean scores between the diabetes group and ASD group

Instrument	ASD group mean score *N*=126	Diabetes group mean score *N*=43	*P* value	Odds ratio	95% confidence interval	
**DASS-21 total**	37.86	41.58	0.460			
**Depression**	12.43	10.81	0.356	0.652	0.325	1.307
**Anxiety**	10.16	10.91	0.642	1.742	0.856	3.544
**Stress**	15.27	14.05	0.473	1.110	0.544	2.267
**PSS-14**	27.61	28.63	0.245			

Table 4 shows the mean scores among the 3 groups for the DASS-21 and PSS-14.

**Table 4:** DASS-21 and PSS-14 mean scores among the ASD group, diabetes group, and control group

Instrument	ASD group mean score *N*=126	Diabetes group mean score *N*=43	Control group mean score *N*=116	*P* value
**DASS-21 total**	37.86	41.58	29.81	0.015
**Depression**	12.43	11.49	9.34	0.042
**Anxiety**	10.16	12.70	7.81	0.007
**Stress**	15.27	17.40	12.66	0.030
**PSS-14**	27.61	28.81	25.87	0.002

The DASS total scores were significantly higher in group 2 and group 1 than in the control group (41.58, 37.86, 29.81, respectively, P=0.015). Depressive symptoms were more prevalent in group 1 than in group 2 and the control group (P=0.042), while anxiety and stress symptoms were higher in group 2 than in group 1 and the control group (P=0.007 and 0.030, respectively). On the PSS-14, group 2 scored 28.81, which was more than group 1 with 27.61 and the control group with 25.87 (P=0.002).

Table 5 shows the prevalence of depression, anxiety, and stress in group 1, group 2, and the control group. The prevalence of depression was higher in group 1 than in group 2 and the control group (57.1, 46.5, and 43.1, respectively) (P=0.046). Anxiety and stress were more prevalent in group 2 than in group 1 and the control group (P=0.034 and P=0.009, respectively).

**Table 5:** Reported distress based on DASS-21 and PSS-14 scores among mothers of all groups

Instrument	ASD group *n*=126		Diabetes group *n*=43		Control group *n*=116		*P* value
	N	%	N	%	N	%	
Dass-21							
Depression							
Yes	72	57.1	20	46.5	50	43.1	0.046
No	54	42.9	23	53.5	66	56.9	
Anxiety							
Yes	62	49.2	27	62.8	50	43.1	0.034
No	64	50.8	16	37.2	66	56.9	
Stress							
Yes	76	60.3	27	62.8	65	56	0.009
No	50	36.7	16	37.2	51	44	
Pss-14	27.61			28.63		25.87	0.004

## Discussion

The findings in this study support the initial hypothesis and other previous reports [3, 5] that depression, anxiety, and stress are higher among mothers of children with ASD and type 1 diabetes than among mothers of children with TD. The impact of having a chronic illness, either ASD or diabetes, in this study on mothers’ health varied: mothers of children with ASD reported more depressive symptoms than the other mothers, while mothers of children with diabetes presented with anxiety and stress symptoms much more than mothers of children with ASD and TD. Depression symptoms may follow the death of relatives or friends, while stress follows other types of stressful events such as having a child with a chronic illness [24]. On the other hand, mothers of children with diabetes have daily worries regarding their children’s blood sugar level and what could adversely impact that level as mothers stated during the interview. These worries continue to operate for a long time due to the chronic nature of the illness and based on the child’s developmental tasks and challenges of growing up [25]. Thus, the persistent feelings of anxiety and worries that mothers of children with diabetes experience exceed those experienced by mothers of children with ASD and TD. The current research showed that levels of depression, anxiety, and stress among the mothers of children with diabetes exceeded those reported by Wiebe et al. [26] and were slightly lower than the rates mentioned by Streisand et al. [10]. The caregiver strain stemming from anxiety about blood glucose monitoring is inversely related to adherence to the management protocol in adolescents [27, 28]. Studies found that the severity of depressive symptoms was associated with maternal employment and strong social support [29] The difference in employment rates among the mothers in the 3 groups was because many mothers in the TD group were recruited from the participating centers’ staff, and many mothers in groups 1 and 2 chose to retire to provide better care to their children. The other explanation was that the mothers in the TD group were more educated and therefore had better chances of finding jobs.

The assessment of parents’ mental health, especially of mothers, during case management of children with ASD and children with diabetes is critical for planning steps of management. It has been shown that presence of high levels of life stress such as having a child with chronic illness are risk factors for poor health outcomes for both parents [12, 28].

### Study limitations

The collected information was obtained from a questionnaire, which is a method known to have a problem of subjectivity. The mothers were not subjected to any structured clinical interview to establish a reliable and valid diagnosis of depression or anxiety. The degree of social impairment of children was not evaluated in this study. Severe social impairment and the presence of behavioral problems among children will add to the mother’s burden and their ability to provide adequate care. Furthermore, the socioeconomic background was not exactly the same among the 3 groups. The mothers of children with TD were more educated and working than the mothers in the ASD and diabetes groups. One might speculate about the effect of these differences on mothers’ coping mechanisms and invariably the level of anxiety and stress. We did not address the duration since the diagnosis was a confounding variable due to the limited sample size. Future study designs should address these limitations and include both parents.

### Clinical implications

Parents’ mental health, especially among mothers, is an important determinant of a better ability to cope with the tasks of taking care of children with behavioral problems, particularly those with ASD and diabetes. In our clinical service context, medical services provided to mothers of children with ASD and diabetes lack a specific provision for individual counseling and support during the process of diagnosis and long-term management. This drawback is probably related to insufficiently trained personnel and the absence of a well-designed strategic follow-up plan. Many clinicians feel that their job is to establish the diagnosis and prescribe appropriate medication. They fail to give enough attention to the mother’s feelings of depression and worries. We suggest that periodically checking mothers/caregivers’ mental health should be a part of any monitoring process. The first step in this direction is to establish special separate services for both children with ASD and children with diabetes; with reduced depression and anxiety, mothers are better able to care for themselves and their children.

## Conclusion

In a case-control study, where cases were mothers of children with ASD or diabetes and controls were from the general population, the prevalence of depression, anxiety, and stress was estimated using DASS-21 and PSS-14 instruments. Mothers of children with either ASD or diabetes scored higher on anxiety, depression, and stress than mothers of children with TD; however, the pattern varied between the ASD and diabetes groups. Mothers of children with ASD showed more depressive symptoms, while mothers of children with diabetes reported a higher prevalence of anxiety and stress. Follow-up monitoring of children with ASD and diabetes should include ways of reducing depression and anxiety among mothers to equip them with a more stable mental health status.

## Data Availability

Not applicable.

## Abbreviations

ADHD: Attention deficit hyperactivity disorder
ADOS: Autism Diagnostic Observation Schedule
ASD: Autistic spectrum disorders
CARS: Childhood Autism Rating Scale
CI: Confidence intervals
DASS-21: Depression, Anxiety and Stress Scale-21 Items
DSM-5: Diagnostic Statistical Manual for Mental Disorders-5
M-CHAT: Modified-Checklist for Autism in Toddlers
OR: Odds ratio
PSS-14: Perceived Stress Scale-14
TD: Typical development

## References

[1] Hayes SA, Watson SL (2012). The impact of parenting stress: a meta-analysis of studies comparing the experience of parenting stress in parents of children with and without autism spectrum disorder. J Autism Dev Disord..

[2] Ansari AM, Jahrami HA (2018). Health of mothers of children with autism spectrum disorders and intellectual disability. Bahrain Med Bull..

[3] Al-Farsi O, Al-Farsi Y, Al-Sharbati M, Al-Adawi S (2016). Stress, anxiety, and depression among parents of children with autism spectrum disorder in Oman: a case–control study. Neuropsychiatr Dis Treat..

[4] Almansour MA, Alateeq MA, Alzahrani MK, Algeffari MA, Alhomaidan HT (2013). Depression and anxiety among parents and caregivers of autistic spectral disorder children. Neurosciences (Riyadh)..

[5] Picardi A, Gigantesco A, Tarolla E, Stoppioni V, Cerbo R, Cremonte M, et al. Parental burden and its correlates in families of children with autism spectrum disorder: a multicentre study with two comparison groups. Clin Pract Epidemiol Ment Health. 2018;14(1):143–76. 10.2174/1745017901814010143. 10.2174/1745017901814010143 PMC6080067 30158998

[6] van Steijn DJ, Oerlemans AM, van Aken MAG, Buitelaar JK, Rommelse NNJ (2013). The reciprocal relationship of ASD, ADHD, depressive symptoms and stress in parents of children with ASD and/or ADHD. J Autism Dev Disord..

[7] Estes A, Olson E, Sullivan K, Greenson J, Winter J, Dawson G, et al. Parenting-related stress and psychological distress in mothers of toddlers with autism spectrum disorders. Brain Dev. 2013;35(2):133–8. 10.1016/j.braindev.2012.10.004. 10.1016/j.braindev.2012.10.004 PMC3552060 23146332

[8] Padden C, James JE (2017). Stress among parents of children with and without autism spectrum disorder: a comparison involving physiological indicators and parent self-reports. J Dev Phys Disabil..

[9] Yaqoob U, Ali Khan M, Khemani L, Ul-Haq F, Rafiq J, Iftikhar AS (2018). Diabetes mellitus in children and its effect on caregivers’ mental health. Cureus..

[10] Streisand R, Mackey ER, Elliot BM, Mednick L, Slaughter IM, Turek J, et al. Parental anxiety and depression associated with caring for a child newly diagnosed with type 1 diabetes: opportunities for education and counseling. Patient Educ Couns. 2008;73(2):333–8. 10.1016/j.pec.2008.06.014. 10.1016/j.pec.2008.06.014 18692342

[11] Jaser SS, Linsky R, Grey M (2014). Coping and psychological distress in mothers of adolescents with type 1 diabetes. Matern Child Health J..

[12] Whittemore R, Jaser S, Chao A, Jang M, Grey M (2012). Psychological experience of parents of children with type 1 diabetes. Diabetes Educ..

[13] Hauenstein EJ, Marvin RS, Snyder AL, Clarke WL (1989). Stress in parents of children with diabetes mellitus. Diabetes Care..

[14] Vartak V, Khadikar A, Khadilka V, et al. Maternal anxiety and competency of mothers of children with type 1 diabetes. Int J Diabetes Dev Ctries. 10.1007/513410-0693-3.

[15] Horsch A, McManus F, Kennedy P, Edge J (2007). Anxiety, depressive, and posttraumatic stress symptoms in mothers of children with type 1 diabetes. J Trauma Stress..

[16] Riahi F, Izadi S (2012). Comparison between the mental health of mothers of children with autism and control group. Iran J Psychiatry Behav Sci..

[17] Fido A, Saad SA (2013). Psychological effects of parenting children with autism prospective study in Kuwait. Open J Psychiatry..

[18] Malallah Al Ansari A, Ali Jahrami H, Ghazi Hafedh R, Mohammed Sharif I (2018). A comparison of the mental health, quality of life, and general functioning of mothers of young children and adolescents with autism. J Bahrain Med Soc.

[19] American Psychiatric Association (2013). Diagnostic and statistical manual of mental disorders.

[20] Taouk M, Lovibond PF, Laube R (2001). Psychometric properties of an Arabic version of the depression anxiety stress scales (DASS-21) report for New South Wales transcultural mental health center.

[21] Ali AM, Ahmed A, Sharaf A, Kawakami N, Abdeldayem SM, Green J. The Arabic version of the Depression Anxiety Stress Scale-21: cumulative scaling and discriminant-validation testing. Asian J Psychiatr. 2017 Dec;30:56–8. 10.1016/j.ajp.2017.07.018 Epub 2017 Jul 18. PMID: 28755630. 10.1016/j.ajp.2017.07.018 28755630

[22] Linn MW (1986). Modifiers and perceived stress scale. J Consult Clin Psychol..

[23] Chaaya M, Osman H, Naassan G, Mahfoud Z (2010). Validation of the Arabic version of the Cohen perceived stress scale (PSS-10) among pregnant and postpartum women. BMC Psychiatry.

[24] Parker G, Paterson A, Hadzi-Pavlovic D (2015). Emotional response patterns of depression, grief, sadness and stress to differing life events: a quantitative analysis. J Affect Disord..

[25] Sullivan-Bolyai S, Deatrick J, Gruppuso P, Tamborlane W, Grey M (2003). Constant vigilance: mothers’ work parenting young children with type 1 diabetes. J Pediatr Nurs..

[26] Wiebe DJ, Gelfand D, Butler JM, Korbel C, Fortenberry KT, McCabe JE (2011). Longitudinal associations of maternal depressive symptoms, maternal involvement, and diabetes management across adolescence. J Pediatr Psychol..

[27] Hansen JA, Weissbrod C, Schwartz DD, Taylor WP (2012). Paternal involvement in pediatric type 1 diabetes: fathers’ and mothers’ psychological functioning and disease management. Fam Syst Health..

[28] Carcone AI, Ellis DA, Naar-King S (2012). Linking caregiver strain to diabetes illness management and health outcomes in a sample of adolescents in chronically poor metabolic control. J Dev Behav Pediatr..

[29] Gjerdingen D, Mcgovirn P, Attonasio L, Johnson PJ, Koshimannil KB. The relationship between maternal depressive symptoms, employment and social support. J AM Board FAM Med. 2014:27(1). 10.3122/Jabfm2014.130126. 10.3122/jabfm.2014.01.130126 PMC3882899 24390890

